# Double-blind, placebo-controlled first in human study to investigate an oral vaccine aimed to elicit an immune reaction against the VEGF-Receptor 2 in patients with stage IV and locally advanced pancreatic cancer

**DOI:** 10.1186/1471-2407-12-361

**Published:** 2012-08-20

**Authors:** Andreas G Niethammer, Heinz Lubenau, Gerd Mikus, Philipp Knebel, Nicolas Hohmann, Christine Leowardi, Philipp Beckhove, Mustafa Akhisaroglu, Yingzi Ge, Marco Springer, Lars Grenacher, Markus W Buchler, Moritz Koch, Jürgen Weitz, Walter E Haefeli, Friedrich H Schmitz-Winnenthal

**Affiliations:** 1 Vaximm, Mannheim, Germany; 2 Clinical Pharmacology and Pharmacoepidemiology University of Heidelberg, Heidelberg, Germany; 3 General, Visceral and Transplantation Surgery University of Heidelberg, Heidelberg, Germany; 4 National Center for Tumor Disease University of Heidelberg, Heidelberg, Germany; 5 Department of Diagnostic and Interventional Radiology University of Heidelberg, Heidelberg, Germany

**Keywords:** DNA vaccine, Oral vaccine, Pancreatic cancer, Cancer vaccine, VEGFR-2, Anti-angiogenesis

## Abstract

**Background:**

The investigational oral DNA vaccine VXM01 targets the vascular endothelial growth factor receptor 2 (VEGFR-2) and uses *Salmonella typhi* Ty21a as a vector. The immune reaction elicited by VXM01 is expected to disrupt the tumor neovasculature and, consequently, inhibit tumor growth. VXM01 potentially combines the advantages of anti-angiogenic therapy and active immunotherapy.

**Methods/Design:**

This phase I trial examines the safety, tolerability, and immunological and clinical responses to VXM01. The randomized, placebo-controlled, double blind dose-escalation study includes up to 45 patients with locally advanced and stage IV pancreatic cancer. The patients will receive four doses of VXM01 or placebo in addition to gemcitabine as standard of care. Doses from 10^6^ cfu up to 10^10^ cfu of VXM01 will be evaluated in the study. An independent data safety monitoring board (DSMB) will be involved in the dose-escalation decisions. In addition to safety as primary endpoint, the VXM01-specific immune reaction, as well as clinical response parameters will be evaluated.

**Discussion:**

The results of this study shall provide the first data regarding the safety and immunogenicity of the oral anti-VEGFR-2 vaccine VXM01 in cancer patients. They will also define the recommended dose for phase II and provide the basis for further clinical evaluation, which may also include additional cancer indications.

**Trial registration:**

EudraCT No.: 2011-000222-29, NCT01486329, ISRCTN68809279

## Background

Angiogenesis contributes to solid tumor growth and metastasis [1]. Compounds like bevacizumab and others, for example small molecules such as sunitinib and axitinib that specifically target the tumor neovasculature have shown efficacy in a range of tumor indications [2-4]. Tumor neovasculature is lined with endothelial cells that are overexpressing vascular endothelial growth factor receptor (VEGFR) 2 and are readily accessible *via* the blood stream [5]. The genetic stability of those cells and their ability to support hundreds of tumor cells per endothelial cell make them a prime target for anti-cancer therapy, be it *via* antibodies, tyrosine kinase inhibitors, or vaccines [6].

Recently, T-cell based immunotherapy has gained some clinical success in prostate cancer and validated the potential of anti-cancer vaccination which was often demonstrated preclinically [7]. Activating the immune system against cancer cells faces multiple challenges. For example, cancerous lesions are often polyclonal and cancer cells have the propensity to mutate. Antigen specific therapy often only results in a selection of non-antigen bearing cells. Further hurdles include tumor encapsulation and loss or down-regulation of MHC molecules. Vaccination approaches that target that the tumor neovasculature should in theory overcome those hurdles.

The trial presented here attempts to combine anti-angiogenic therapy and vaccination, targeting VEGFR-2 using a new vaccine (VXM01). Hypothetically, vaccination with VXM01 should lead to breakdown of existing tumor vasculature and support the development of an immune memory against proliferating endothelial cells.

In 2002, we published a preclinical study combining anti-angiogenic therapy and vaccination for the first time [8]. Vaccinating mice against the VEGFR-2 induced strong resistance against a variety of different tumor challenges such as melanoma, colon cancer, and lung cancer, both in prophylactic as well as therapeutic experimental settings. These effects were long-lived and mediated by cytotoxic T-cells. Surprisingly, a slight delay in wound healing was the only toxicity that was observed.

To our knowledge, this is the first clinical trial of an oral cancer vaccine. In addition, this vaccine has the potential to be effective against multiple tumor types.

## Methods

### Preclinical efficacy assessment

The efficacy and safety of this approach in animals has been validated multiple times by us and others [8-10]. Further, own unpublished experiments showed an activity of this vaccine in two different models of pancreatic cancer.

VXM01, the vaccine used in this trial, is a humanized version of the anti-VEGFR-2 vaccine previously tested in mice. It encodes the human full-length VEGFR-2 and uses the licensed *Salmonella typhi* strain Ty21a instead of *Salmonella typhimurium* as a carrier. The vaccine is assumed to lead to VEGFR-2 protein expression in monocytes and dendritic cells after entry of VXM01 in the Peyer’s patches *via* M cells of the gut, and internalization by antigen-presenting cells followed by translation of the encoded DNA [11,12].

### Preclinical safety assessment

Preclinical toxicity studies in mice included, but were not restricted to a single dose toxicity study in mice conducted with the human vaccine VXM01. As VXM01 is specific for the human host, the study of the human vaccine in mice focused on possible effects of process-related impurities and related signs and symptoms of possible relevance for cardiovascular, respiratory, or central nervous system impairment. In order to investigate the toxicity profile of an anti-VEGFR-2 T-cell response, a repeated dose toxicity study was conducted using the murine analog construct of VXM01 which induced a dose-dependent T-cell response in mice. In accordance to our previous observations, no treatment-related deaths and no toxicologically important clinical signs were observed throughout these studies, which were conducted according to Good Laboratory Practice (GLP).

The vector *Salmonella typhi* Ty21a used here is a live, attenuated bacterial carrier that allows for the oral delivery of the vaccine VXM01. It is itself an approved vaccine against typhoid fever (Vivotif®, Crucell, formerly Berna Biotech Ltd., Switzerland) that has been extensively tested and has demonstrated its safety regarding patient toxicity as well as transmission to third parties [13,14]. VXM01 is classified as a gene transfer medicinal product and subject to the respective guidance and regulations [15].

This study has been approved by the German regulatory agency, the Paul-Ehrlich-Institute (PEI). In accordance with the Declaration of Helsinki [16], the German Drug Law (Arzneimittelgesetz [AMG]) [15], the German Good Clinical Practice guideline (GCP-V) [17], and the Note for Guidance on Good Clinical Practice (GCP) [18], the study was presented to the responsible Ethics Committee of the Medical Faculty of the University of Heidelberg and the PEI. The opinion of the ethics committee and the authorization of the PEI were obtained prior to any study-related procedures. The recruiting study center is the Clinic of General Surgery; drug exposure is done and patients are supervised at the ISO-certified Clinical Research Unit (KliPS) of the Department of Clinical Pharmacology and Pharmacoepidemiology, Heidelberg University Hospital, Heidelberg, Germany. Study patients will be hospitalized for ten days in KliPS during the sensitive administration period of the vaccine.

### Study description

This is a monocenter, placebo controlled, double blind dose escalation study of the experimental vaccine VXM01 in patients with advanced inoperable or stage IV pancreatic cancer. The vaccine will be given as add-on to a standard of care gemcitabine treatment.

### Study objectives

The objectives are to examine the safety and tolerability, and immunological and clinical responses to the investigational anti-VEGFR-2 vaccine VXM01, as well as to identify the maximum tolerated dose (MTD) of VXM01. The MTD is defined as the highest dose level at which less than two of up to six patients under VXM01 treatment experience a dose-limiting toxicity (DLT).

Primary endpoints for safety and tolerability are as follows: Number of DLTs defined as any adverse event (AE) related to study drug of grade 4 or higher, or grade 3 or higher for gastrointestinal fistula, diarrhea, gastrointestinal perforation, multi-organ failure, anaphylaxis, any auto-immune disorder, cytokine-release syndrome, intestinal bleeding, renal failure, proteinuria, thromboembolic events, stroke, heart failure, or vasculitis according to the National Cancer Institute Common Terminology Criteria for Adverse Events (CTCAE) [19].

Secondary endpoints, which assess the efficacy of the experimental vaccine to elicit a specific immune response to VEGFR-2, include the number of immune positive patients.

A further secondary endpoint will be the clinical response: Tumor staging according to the response evaluation criteria in solid tumors (RECIST) [20], overall response rate, progression free survival, overall survival, and changes in tumor perfusion. Tumor perfusion will be determined by dynamic contrast-enhanced Magnetic Resonance Imaging (DCE-MRI) [21] on a 1.5 Tesla system (Magnetom Aera, Siemens, Erlangen, Germany).

### Investigational products

VXM01 has been manufactured according to Good Manufacturing Practice (GMP) and is given in a buffered solution. The placebo control consists of isotonic sodium chloride solution.

### Patient selection and study design

This study will include a maximum of 45 patients with either locally advanced and inoperable or stage IV pancreatic cancer. The eligibility criteria are summarized in Table 1.

**Table 1:** Eligibility Criteria

	
**Inclusion Criteria**	
1	Written informed consent, signed and dated
2	Locally advanced, inoperable and stage IV pancreatic cancer patients according to UICC based on diagnostic imaging using computer-tomography (CT) or histological examinations
3	Male or post-menopausal female
4	Age ≥18 years
5	Chemotherapy naïve within 60 days before screening visit except gemcitabine treatment
6	Karnofsky index >70
7	Life expectancy >3 months
8	Adequate renal, hepatic, and bone marrow function
9	Absolute neutrophil count >1500/μL
10	Hemoglobin >10 g/dL
11	Platelets >75000/μL
12	Prothrombin time and international normalized ratio (INR) <1.5 times upper limit of normal (ULN) (except under anticoagulant treatment)
13	Aspartate aminotransferase <4 times ULN
14	Alanine aminotransferase <4 times ULN
15	Total bilirubin <3 times ULN
16	Creatinine clearance estimated according to Cockcroft-Gault > 30 mL/min
17	Proteinuria <1 g protein on 24 h urine collection
**Exclusion Criteria**	
1	State after pancreas resection (complete or partial)
2	Resectable disease
3	Drug trial participation within 60 days before screening visit
4	Other previous or current malignancy except basal or squamous cell skin cancer, in situ cervical cancer, or any other cancer from which the patient has been disease-free for <2 years
5	Prior vaccination with Ty21a
6	Cardiovascular disease defined as:
	Uncontrolled hypertension (systolic blood pressure >160 mmHg or diastolic blood pressure >100 mmHg)
	Arterial thromboembolic event within 6 months before randomization including:
	- Myocardial infarction
	- Unstable angina pectoris
	- Cerebrovascular accident
	- Transient ischemic attack
7	Congestive heart failure New York Heart Association grade III to IV
8	Serious ventricular arrhythmia requiring medication
9	Clinically significant peripheral artery disease > grade 2b according to Fontaine
10	Hemoptysis within 6 months before randomization
11	Esophageal varices
12	Upper or lower gastrointestinal bleeding within 6 months before randomization
13	Significant traumatic injury within 4 weeks before randomization
14	Non-healing wound, bone fracture or any history of gastrointestinal ulcers within three years before inclusion, or positive gastroscopy within 3 months before inclusion
15	Gastrointestinal fistula
16	Thrombolysis therapy within 4 weeks before randomization
17	Bowel obstruction within the last 30 days before screening visit
18	Liver cirrhosis ≥ grade B according to Child-Pugh Score-Classification
19	Presence of any acute or chronic systemic infection
20	Radiotherapy within 4 weeks before randomization
21	Major surgical procedures, or open biopsy within 4 weeks before randomization
22	Fine needle aspiration within 7 days before randomization
23	Chronic concurrent therapy within 2 weeks before and during the double-blind study period with:
	- Corticosteroids (except steroids for adrenal failure) or immunosuppressive agents
	- Antibiotics
	- Bevacizumab
	- Any epidermal growth factor receptor inhibitor
	- Chemotherapy except gemcitabine before Day 10
224	Multi-drug resistant gram-negative germ
25	Pregnancy
26	Lactation
227	Inability to comply with study and/or follow-up procedures
28	History of other disease, metabolic dysfunction, physical examination finding, or clinical laboratory finding giving reasonable suspicion of a disease or condition that contraindicates the use of an investigational drug or that might affect the interpretation of the study results or render the patient at high risk for treatment complications
29	Women of childbearing potential
30	Any history of drug hypersensitivity
31	Any condition which results in an undue risk for the patient during the study participation according to the investigator

Male and postmenopausal female patients will be enrolled in this study. However, differences between the two genders will not be investigated. The average survival time of the patients participating in this trial is under 6 months. However, the follow-up period for the patients as defined per protocol is up to 24 months. The study treatment might confer clinical benefit and is given first-line as an add-on to standard of care. Taking further into account other factors, among them the multiple primary and secondary pharmacodynamic preclinical studies, the risk-benefit analysis is assumed to have a favorable result for the patient population selected.

The patient population and the study design were discussed in National Scientific Advice meetings with the PEI (Langen, Germany) and the Medical Products Agency (Uppsala, Sweden) and were considered adequate to achieve the study objectives. The first administration of a gene transfer medicinal product cannot be justified in healthy volunteers.

The starting dose consists of a solution containing 10^6^ colony forming units (CFU) of VXM01 or placebo. This VXM01 dose was chosen for safety reasons and is assumed to be below the minimal effective dose to elicit an immune response. For comparison, one dose of Typhoral®, the licensed vaccine against typhoid fever, contains 2x10^9^ to 6x10^9^CFU of *Salmonella typhi* Ty21a, equivalent to approximately thousand times the VXM01 starting dose [22]. The dose will be escalated in factor-of-ten logarithmic steps, which appears to be justified for a live bacterial vaccine.

Complying with guidelines of first in human trials [23], the patients of one dose group will be treated in cohorts. The first administration of VXM01 in any dose group will be given to one patient only accompanied by one patient receiving placebo. The second cohort of each dose group consists of two patients receiving VXM01 and one patient receiving placebo. This staggered administration with one front-runner, i.e. only one patient receiving VXM01 first, serves to mitigate the risks [23].

**Figure 1:**
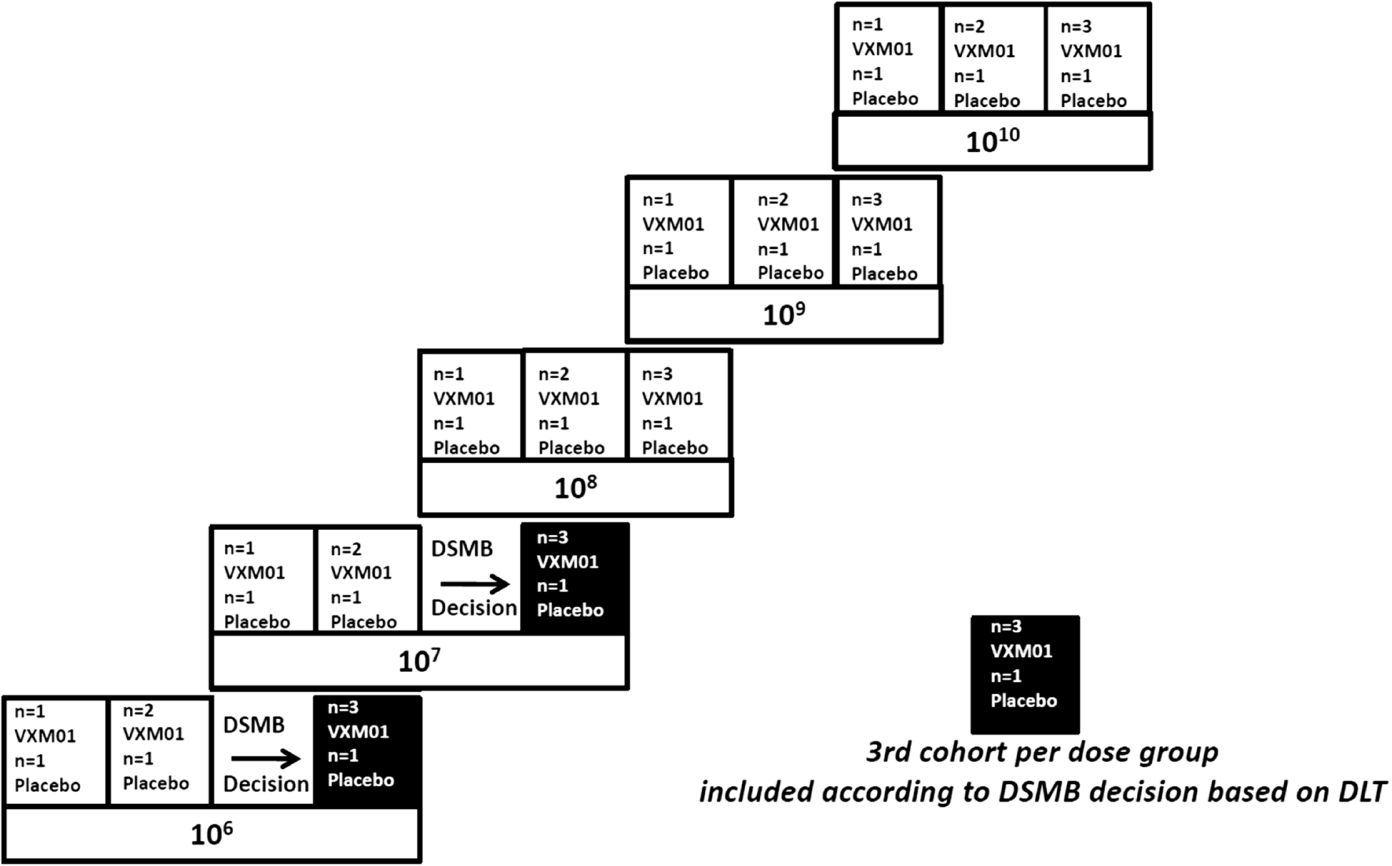
Dose escalating design.

A third cohort of patients (three receiving VXM01 and one receiving placebo) will be included in the 10^8^, 10^9^, and 10^10^ dose groups. This approach minimizes exposure to VXM01 doses assumed to be sub-therapeutic. The third cohort and the first two cohorts of the next higher treatment group may be treated in parallel based on a clearly defined randomization strategy. This strategy allows for recruitment of available patients and avoids selection bias for patients treated in parallel in the lower and higher dose group. In the 10^6^ and 10^7^ dose groups, a third cohort of patients will be included only if one patient out of the initial three patients receiving VXM01 of the respective dose group experiences a DLT and requires confirmation by a decision of the Data Safety Monitoring Board (DSMB). The study design is depicted in Figure 1.

The environmental risk inherent to an oral vaccine is the potential of excretion to the environment and subsequent vaccination of people outside the target population. All study patients will be confined in the study site (KliPS) for the period during which vaccinations take place plus three additional days. All feces of study patients will be collected and incinerated. Body fluids and feces samples will be investigated for VXM01 shedding. As documented in the literature Ty21a, the bacterial carrier of VXM01 was not excreted up to a dose of 10^9^ cfu After administration of a dose of 10^10^ cfu of Ty21a fecal excretion was observed until two days after vaccination [24].

Hygienic precautions will be applied to protect study personnel from accidental uptake. Study personnel will be trained specifically for this aspect of the study.

Patients will only be discharged from hospital if they test negative for excretion of the vaccine after the last administration of the study drug. In case a patient tests positive for excretion after the last administration, an antibiotic decontamination of the gastrointestinal tract will be conducted before the patient is discharged. Excretion will be followed up until results are negative. These measures appear to be justified and sufficient to protect the environment and study personnel from exposure to VXM01 until the shedding profile has been elucidated.

**Figure 2:**
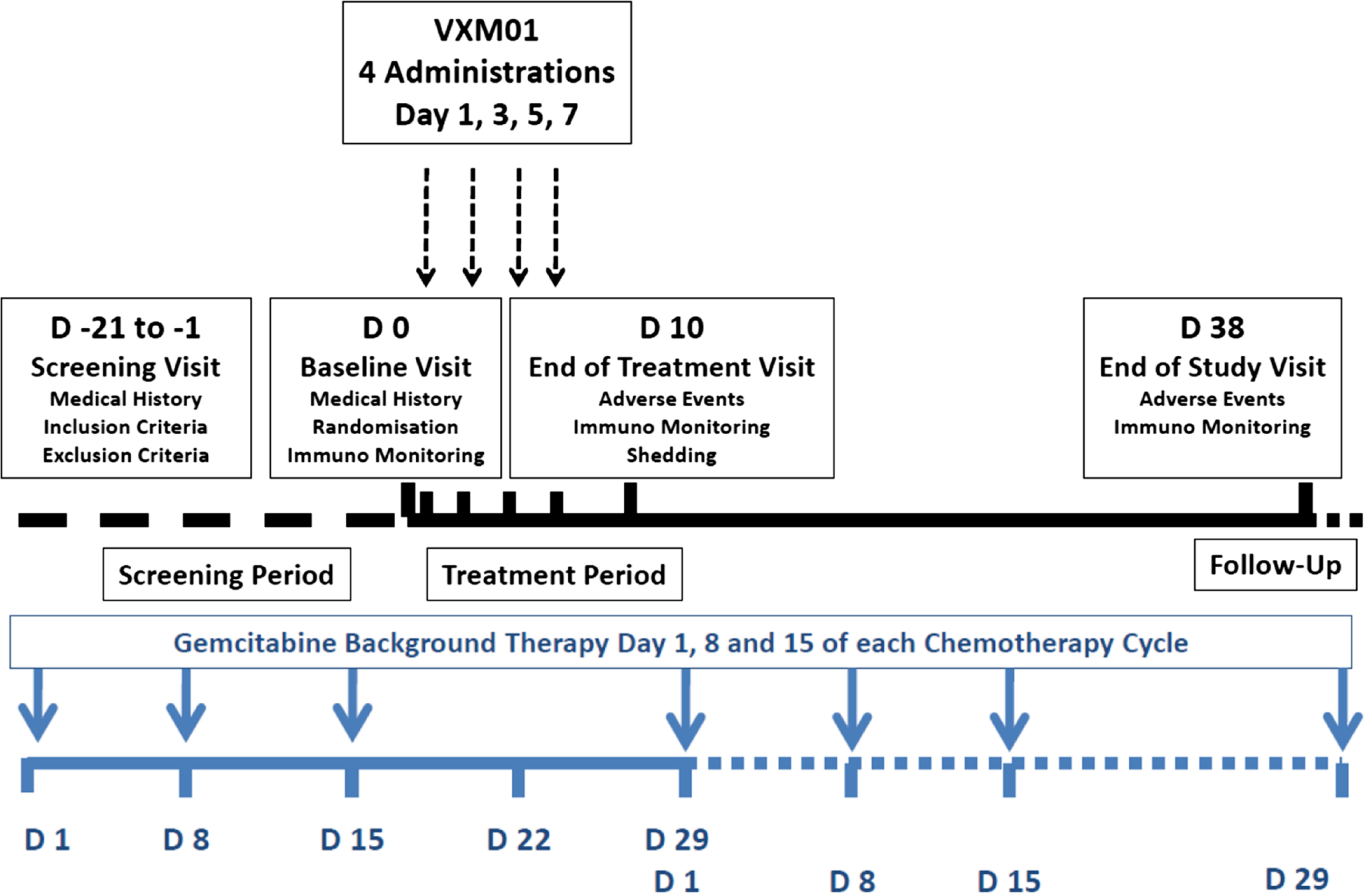
Overall Study Scheme.

VXM01 will be applied in parallel to the gemcitabine background therapy as shown in Figure 2 (overall study scheme). In brief, gemcitabine is given on days 1, 8, and 15 of a 28 days chemotherapy cycle. The vaccine will be given four times on days 1, 3, 5, and 7, starting three days after the last dose of gemcitabine. The double blinded phase of the study will end 31 days after the last patient has received the last administration.

## Discussion

This study aims to find a safe and effective dose of the experimental oral vaccine VXM01 targeting the human VEGFR-2 for use during further investigation in a phase II clinical trial. Despite years of failure, a first active immune therapy, Dendreon’s Provenge, was efficacious in prostate cancer, and as a consequence has been approved by the FDA [25]. Several more tumor vaccines are now in the midst or entering late-stage development [26]. However, all these approaches are targeting so-called tumor-associated antigens (TAA). For example, vaccines targeting MUC-1 (stimuvax) and the TAA Mage-A3 are currently under development in phase III clinical trials [27,28].

VXM01 represents a novel strategy by targeting not a tumor cell-resident antigen, but a tumor stroma-resident antigen, overexpressed by non-malignant endothelial cells of the tumor neovasculature. A Japanese group has recently published a phase I study, implementing a single VEGFR-2-derived peptide administered in weekly intervals *via* the subcutaneous route of administration, thus following a similar approach, but restricting it to a certain HLA type [29].

By targeting genetically stable and easily accessible endothelial cells, this product aims to overcome limitations encountered previously by vaccines targeting tumor cells directly, such as to tumor-cell heterogeneity, MHC-loss, immunosupression on a cellular level and tumor encapsulation as well as physiological barriers such as the blood brain barrier. Furthermore, since the therapeutic target is independent of the tumor type, the vaccine may potentially be active against a variety of different solid malignancies. The product represents a patient-independent, “off-the-shelf” oral vaccine, which can be stored and distributed to the clinical sites for use. While anti-angiogenic therapy, either *via* small molecules or *via* antibodies, has already been proven to be effective, our approach differs significantly by activating the patient’s own immune system against tumor neovasculature and is as such potentially creating a T-cell memory effect that provides long-term efficacy. Studies with bevacizumab in colon and ovarian cancer suggest that continued anti-angiogenic pressure is required to maintain beneficial treatment effects in the long term [30-32].

Should adverse events occur that resemble hypersensitivity reactions mediated by histamine, leukotrienes, or cytokines, treatment options for fever, anaphylaxis, blood pressure instability, bronchospasm, and dyspnoea are available. Treatment options in case of unwanted T-cell derived autoaggression are derived from standard treatment schemes in acute and chronic graft vs. host disease applied after stem cell transplantation. Cyclosporin and glucocorticoids are proposed as treatment options.

In the unlikely case of systemic *Salmonella typhi* Ty21a type infection, appropriate antibiotic therapy with fluoroquinolones including ciprofloxacin or ofloxacin is recommended [33]. Bacterial infections of the gastrointestinal tract are to be treated with rifaximin.

For this phase I trial (advanced or stage IV pancreatic cancer patients) a patient population with dismal prognosis and the relatively gentle standard of care with regard to immunosuppression was chosen. Co-regimes of gemcitabine with tumor vaccination have even been reported to be synergistic [34,35]. In addition, specific T-cell activation can be measured in this patient setting and may give an early indication of potential effectiveness of the vaccine VXM01. By including a placebo control in the present trial, we will gain further knowledge on specific safety issues related to the active vaccine vs. the background treatment. In addition, the pooled placebo patients will serve as a sound comparator in order to assess specific immune activation and other signs of clinical efficacy. If and when moving into phase II, a different patient entity with a longer life expectancy can be envisaged depending on the observed safety profile. Such studies will also include tumor types that have shown to be more susceptible to anti-angiogenic treatment.

We recognize the limitations inherent with a single center study and hope to partially address any bias by the introduction of the blinded placebo patients and an independent unblinded DSMB, which is without direct patient access. Further, the lack of a clear expectation as to what constitutes a sufficient positive immune reaction may be viewed as problematic. However, the read-out allows for a proof of a successful specific immune reaction. We have set predefined thresholds as to the positivity of our immunological assays. As to how far this can be correlated with dose and/or patient’s response remains to be seen.

## Conclusions

VXM01 has the potential to target a variety of tumor types and to overcome multiple hurdles encountered by other present cancer vaccine approaches. A tempting vision is the possibility of combining our vaccine with a multitude of other anti-cancer and immune-modulatory agents, provided that the toxicity profile encountered in humans will allow so.

The results of the here presented study will guide us to either modify our approach or to move forward into the next steps of clinical development.

## Competing interests

This study is sponsored by VAXIMM.

AG Niethammer holds stock options of VAXIMM.

H. Lubenau is an employee of VAXIMM and holds stock options. M. Springer is an employee of VAXIMM and holds stock options.

## Authors’ contributions

AGN was involved in preclinical studies, the design of the trial, and in the drafting of the protocol and the manuscript. HL was involved in the design, submission, implementation, coordination of the trial, and in the drafting of the protocol and the manuscript. MS was coordinating the preclinical toxicity studies, the setup of the immune monitoring and biodistribution part of the trial and contributed to the drafting of the manuscript. LG has developed the MR protocol for DCE-MRI and is responsible for imaging evaluation of the functional MR dataset and post processing of the trial and contributed to the drafting of the manuscript. MK is involved in the supervision of the trial and revised the manuscript. PK was involved in the implementation and coordination of the trial. MWB is involved in the supervision of the trial and revised the manuscript. JW is involved in the supervision of the trial and revised the manuscript. WEH was involved in the design, implementation, submission of the trial, and in the drafting of the protocol and the manuscript. GM contributed to the design and coordination of the trial, and to the drafting of the protocol and the manuscript. NH was involved in the preparation and conduct of the trial. FHSW was involved in preclinical studies, the design, implementation, submission of the trial, in the drafting of the protocol, the manuscript and is the PI of the running trial. All authors read and approved the final manuscript.

## Pre-publication history

The pre-publication history for this paper can be accessed here:

http://www.biomedcentral.com/1471-2407/12/361/prepub
